# Effects of Taijiquan on glucose and lipid metabolism in middle-aged and elderly diabetic patients: A protocol for systematic review and meta-analysis

**DOI:** 10.1097/MD.0000000000024433

**Published:** 2021-01-29

**Authors:** Ya-Nv Liu, Lin Wang, Xin Fan, Shijie Liu, Qi Wu, You-Lin Qian

**Affiliations:** a Department of Physical Education, Wuhan University of Technology, Wuhan; b School of Physical Education, Hubei Normal University, Huangshi; c School of Physical Education and Sport Training, Shanghai University of Sport, Shanghai; d School of Physical Education, Hubei Minzu University, Enshi, China; e Graduate school, Adamson University, Manila, Philippines.

**Keywords:** diabetes, glycolipid metabolism, meta-analysis, Taijiquan

## Abstract

**Background:**

The improvement effect of exercise on diabetes mellitus has been widely recognized. Taijiquan, as a popular exercise mode for middle-aged and elderly people, is not clear about its effect on glucose and lipid metabolism in elderly diabetic patients. In this paper, the influence of Taijiquan on glucose and lipid metabolism in middle-aged and elderly diabetic patients was studied by using a meta-analysis method, to provide evidence for the clinical promotion of Taijiquan to improve glucose and lipid metabolism in diabetic patients.

**Methods:**

Computer system search and manual search were conducted respectively in web of science, PubMed, Cochrane Library, EMBASE, Google Scholar, China National Knowledge Infrastructure, Wanfang Database, VIP from the inception to August 1, 2020. Randomized controlled trials of the application of Taijiquan in middle-aged and elderly diabetic patients were collected.

**Results:**

The current study is a systematic review and meta-analysis program with no results. Data analysis will be completed after the program.

**Conclusion:**

This review aims to study the effect of Taijiquan on the glucose and lipid metabolism of middle-aged and elderly diabetic patients, objectively evaluate the effect of Taijiquan on the glucose and lipid metabolism of middle-aged and elderly diabetic patients, and provide scientific basis for clinical exercise intervention in middle-aged and elderly diabetic patients.

**Protocol registration number:**

INPLASY2020120107

## 1 Introduction

### 1.1 Description of the condition

Diabetes is a group of metabolic diseases characterized by chronic blood glucose (blood sugar) increase, with the characteristics of high disability, high mortality, and high complications.^[1]^ The main cause of diabetes is the defect of insulin secretion in the body, which leads to insufficient insulin secretion or the secreted insulin has no biological effect on the receptor, which in turn leads to the disorder of protein, fat, carbohydrate, and other substances in the body. The onset of diabetes may lead to pathological changes in organs such as the heart and kidneys, leading to various diseases such as diabetic nephropathy and diabetic liver disease. In recent years, the number of patients with diabetes has been increasing. The International Diabetes Federation report shows that in 2019, approximately 463 million adults aged 20 to 79 have diabetes worldwide, of which 135.6 million elderly people have diabetes, with a prevalence rate of 19.3%, The number of elderly diabetic patients in China ranks first in the world. https://diabetesatlas.org/en/sections/demogr-aphic-and-geogra-phic-outline.html. The National Diabetes Epidemiological Survey found that the prevalence of diabetes in the 25 to 34-year-old population in 2007 increased by 8 times compared with 1994, and diabetes is gradually showing a younger trend,^[2]^ so the research on diabetes cannot be limited to the elderly population. Based on the high incidence and fatality rate of diabetes and the heavy burden brought to society and families, it is urgent to find an effective method to treat diabetes.

### 1.2 Description of intervention

Taijiquan is one of the most popular types of martial arts. It originated from Qi's “32 potentials”, spread in the “gun boxing”, and was formed in the “13 potentials”.^[3]^ It runs through the classical philosophy and traditional Chinese medicine theory in the boxing, and accumulates the guidance and techniques of breathing, thus achieving the characteristics of gentle, gentle and flexible movements of Taijiquan. In order to regulate the 5 ZANG organs, balance Yin and Yang, and calm down and calm down the god, Taijiquan emphasizes the fitness effect of relaxation, softness and slowness, opening and closing orderly, hardness and softness. Studies have shown that Taijiquan can improve the body's immunity, improve the body's ability to resist diseases, and prevent the occurrence of diseases.^[4,5,6]^ In recent years, the use of Taijiquan to improve chronic diseases has gradually increased, such as using Taijiquan to improve hypertension,^[7]^ coronary heart disease,^[8]^ chronic obstructive pulmonary disease,^[9]^ and using Taijiquan to interfere with middle-aged and elderly diabetic patients It also shows an increasing trend,^[10,11,12,13]^ but the research conclusions are not completely consistent. Therefore, this study uses Taijiquan to systematically evaluate and meta-analyze randomized controlled trials (RCTs) of glucose and lipid metabolism in middle-aged and elderly diabetic patients to objectively evaluate Taijiquan to The influence of glucose and lipid metabolism in middle-aged and elderly diabetic patients provides a scientific basis for clinical exercise intervention in middle-aged and elderly diabetic patients.

### 1.3 Objective of this study

Meta-analysis is used to explore the effect of Taijiquan on the metabolism of glucose and lipid in middle-aged and elderly diabetic patients.

## 2 Methods

### 2.1 Registration

This system evaluation program has been registered in the International Registration System Evaluation and Meta-Analysis Program Platform, the registration number is INPLASY2020120107. In this article, the Cochrane Handbook (version 5.1.0, http://www.cochranehandbook.org) for the systematic review of interventions will be used as a guide for the systematic review. This review plan will be reported in strict accordance with the list of preferred reporting items and meta-analysis plans for system review.^[14]^

### 2.2 Eligibility criteria

We will include studies according to the criteria outlined below

#### 2.2.1 Study designs

Only RCTs including monotherapy therapy of Taijiquan. Exclude research reviews, cross-sectional studies, conferences, observational studies, and case reports.

#### 2.2.2 Types of participants

The test population is clinically diagnosed middle-aged and elderly diabetic patients, regardless of gender differences, regional differences and ethnic background, but if the patient is seriously ill and cannot participate in the experimental intervention or suffers from serious other diseases at the same time (for example: heart disease, cerebral infarction, Alzheimer disease) was excluded from the subjects.

#### 2.2.3 Types of interventions

Taijiquan has undergone a long process of evolution and contains multiple types. Therefore, this study accepts all types of single Taijiquan interventions, and is not limited by the type, frequency, duration, location, and intensity of the intervention. If Taijiquan combined with other forms of exercise therapy will be excluded.

#### 2.2.4 Types of controls

The control group should adopt one of the following treatment methods: waiting list, routine nursing, routine exercise, non-intervention, etc. In addition, exercises related to or similar to Taijiquan should be excluded as the control group, so as not to affect the accuracy of the research results.

#### 2.2.5 Types of outcome measures

The research results of this system review include the following aspects: fasting blood glucose, glycosylated hemoglobin, total cholesterol, triglycerides, high-density lipoprotein cholesterol, and low-density lipoprotein cholesterol. The measurement of the above 6 results requires fasting blood samples. The plasma separated from the blood samples is used to measure fasting blood glucose, and the serum separated from the blood samples is used to measure glycosylated hemoglobin, triglycerides, total cholesterol, high density lipoprotein cholesterol, and low density. Lipoprotein cholesterol.

### 2.3 Search methods

#### 2.3.1 Information sources

In order to avoid missing any documents related to this system review, we selected 5 English databases (Web of Science, PubMed, The Cochrane Library, EMBASE, Google Scholar) and 3 Chinese databases (China National Knowledge Infrastructure, VIP, Wanfang) search for related research, all Chinese and English documents published from the date of establishment to August 1, 2020, are not restricted by race, region, or gender. At the same time, the search should also cover the World Health Organization international clinical trials The registration platform machine registers the network to obtain unpublished and other research that is ready for publication. In addition, relevant literature is screened from the references of included studies and related reviews to ensure that the studies are fully included.

#### 2.3.2 Search strategies

The search term is Tai Chi or Tai Ji or Tai Chi exercise or Tai Ji Chuan or Tai Ji Quan or Tai Chi Chuan or Tai Chi Quan or and Diabetes or Diabetes or Type 2 Diabetes or Type 1 Diabetes Mellitus. Two researchers independently conducted the literature. search for. This research will take Web of Science as an example to show the first draft of the search strategy (Table 1).

**Table 1:** Web of Science search strategies.

Order	Search terms
#1	Diabetes or diabetes mellitus or type 2 diabetes mellitus or type 1 diabetes mellitus
#2	Tai Chi or Tai Ji or Tai Chi exercise or Tai Ji Chuan or
	Tai Chi Quan or Tai Chi Chuan or Tai Chi Quan or Taichi
	or Taichiquan
#3	#1 and #2

### 2.4 Data collection

#### 2.4.1 Study selection

**Figure 1:**
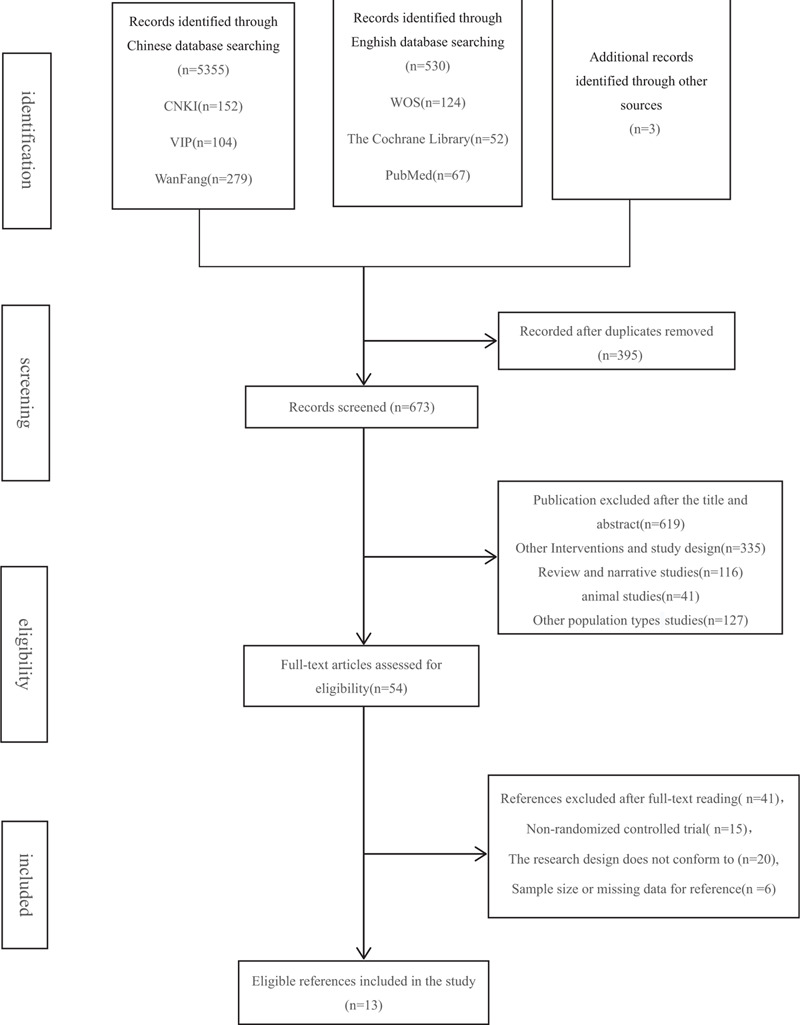
Flow of study selection.

The literature inclusion of this study was independently completed by 2 researchers according to the inclusion and exclusion criteria of the literature. By gradually reading the title, abstract, and full text, the literature that was not related to the study was screened out. If there is any disagreement during the selection of the literature, the 3 researchers will decide whether to include it after discussion, and finally determine the qualified literature for inclusion in this study. The research selection flowchart is shown in Figure 1.

#### 2.4.2 Data extraction

The data extraction of qualified studies was completed independently by 2 researchers. The basic information extracted includes: the name of the first author, the year of publication, the characteristics of the test, the intervention methods, the intervention period, the intervention frequency, and the type of outcome indicators. If 2 researchers encounter differences in the process of extracting basic information, the 3 researchers will discuss and resolve them together.

### 2.5 Data analysis

#### 2.5.1 Data synthesis

Quantitative analysis was performed by meta-analysis using Cochrane collaboration Stata 14.0 software (Stata, Inc., Texas, USA). Continuous variables are described by the average difference between groups and a 95% confidence interval.

#### 2.5.2 Assessment of heterogeneity and publication bias

The X^2^ test is used to test the heterogeneity among the studies. When the included studies are statistically homogeneous (I^2^ < 50%), the fixed-effects model is used for Meta analysis; when the included studies are statistically heterogeneous When it is larger (I^2^ ≥ 50%), the random effects model is used for meta-analysis. When I^2^ is greater than 75%, it is considered to have a high degree of heterogeneity. The source and control of heterogeneity are analyzed by subgroup analysis. The publication bias was assessed using a funnel chart.

#### 2.5.3 Subgroup analysis and sensitivity analysis

The subgroup analysis will be based on exercise period (≤12week; >12week), exercise frequency (≤3 times; >3 times), single exercise time (≤30 minutes; >30 minutes), sports events (Yang style Taijiquan; Chen style Taijiquan) Boxing; mixed Taijiquan), gender (more women than men; women less than men) are further stratified. In order to explore whether the heterogeneity among the included studies is caused by a single study, a sensitivity analysis of the heterogeneity was carried out and the stability of the overall effect size was evaluated by eliminating the individual studies one by one.

#### 2.5.4 Grading the quality of evidence

Two researchers evaluated the quality of the included qualified literature according to the Physiotherapy Evidence Database (PEDro) scale.^[15]^ The PEDro scale has 11 dimensions: eligibility criteria evaluation, randomization, allocation hiding, similar baseline, participant blinding, instructor blinding, evaluator blinding, retention rate of over 85%, treatment intention analysis, comparison between groups, point measurement, and variability measurement.^[16]^ Satisfying one of the indicators is counted as 1 point, otherwise it will be counted as 0 points. Since the first item of the PEDro scale is not included in the total score, the total score of the scale is 10 points, and the total score ≥ 7 is high quality, and 5 to 6 points. Medium quality, ≤4 is classified as low quality,^[17,18]^ and only studies with a score of 5 or more are included.

## 3 Discussion

The International Diabetes Federation pointed out that a healthy diet and regular physical exercise play a major role in preventing type 2 diabetes, and regular physical exercise is essential to control blood sugar. https://www.idf.org/aboutdiabetes/type-2-diabetes.html. Part of the exercise is often recommended to diabetic patients as one of the measures to help improve diabetes metabolism. Some researchers have used meta-analysis methods to explore the effects of exercise on the glucose and lipid metabolism of diabetic patients. For example, De Nardi et al used meta-analysis methods to explore the effect of high-intensity intermittent exercise on improving glycosylated hemoglobin in patients with type 2 diabetes. High-intensity intermittent exercise can effectively improve glycosylated hemoglobin in patients with type 2 diabetes;^[19]^ Yang Jipeng and others found that Health Qigong Ba Duan Jin can reduce glycosylated hemoglobin, fasting blood sugar, total cholesterol in type 2 diabetes, and increase high-density lipoprotein cholesterol.^[20]^ At present, there are still differences in the research conclusions of using Taijiquan to improve the glucose and lipid metabolism of diabetic patients, and the use of Taijiquan exercise to intervene in middle-aged and elderly diabetic patients has not yet been reported. This study uses Taijiquan for middle-aged and elderly patients. RCTs of glucose and lipid metabolism in diabetic patients were systematically reviewed and meta-analyzed to objectively evaluate the effect of Taijiquan on the glucose and lipid metabolism of middle-aged and elderly diabetic patients, and provide scientific basis for clinical exercise intervention in middle-aged and elderly diabetic patients.

Advantages of the research: Firstly, the included literature in this study is relatively comprehensive. Not only did a comprehensive search of the Chinese and English electronic databases, but also the included literature and references of the same type of reviews to ensure the comprehensiveness of the included literature, the literature screening, quality evaluation, and data extraction of this study are all independently completed by 2 researchers. If there is a disagreement during the period, the 3 researchers will make a joint decision after discussion to ensure the consistency of the reviewers; finally the type of articles included in this study is limited to articles from RCTs, which directly improves the quality of this review.

Limitations of the study: first of all, the middle-aged and elderly diabetic patients included in the study have a large difference in the duration of illness, and the drug treatments they receive during the intervention period are also different, which may be one of the reasons for the heterogeneity. Secondly, in this study, the control group received inconsistent intervention measures. Some study control groups also performed aerobic exercise while receiving drug treatment. Different intervention measures in the control group had a certain impact on the results of this study. Third, although the search strategy used in this study is more comprehensive when searching for documents, due to language constraints, there may be some omissions in the study, which may cause bias.

## Author contributions

**Conceptualization:** Ya-nv Liu, Lin Wang, Xin Fan

**Data curation:** Shijie Liu, Qi Wu, You-Lin Qian

**Formal analysis:** Ya-nv Liu, Lin Wang, Xin Fan

**Funding acquisition:** Lin Wang.

**Investigation:** Ya-nv Liu, Lin Wang, Xin Fan, Shijie Liu

**Methodology:** Ya-nv Liu, Lin Wang, Xin Fan

**Software:** Ya-nv Liu, Lin Wang, Xin Fan

**Supervision:** YaNv Liu, Lin Wang, Xin Fan, Shijie Liu.

**Writing – original draft:** Ya-nv Liu, Lin Wang, Xin Fan

**Writing – review & editing:** Ya-nv Liu, Lin Wang, Xin Fan, Shijie Liu, Qi Wu, You-Lin Qian

## Abbreviations

Abbreviations: PEDro = Physiotherapy Evidence Database, RCT = randomized controlled trials.
